# Cell fusing agent virus (*Flavivirus*) infection in *Aedes aegypti* in Texas: seasonality, comparison by trap type, and individual viral loads

**DOI:** 10.1007/s00705-020-04652-0

**Published:** 2020-05-21

**Authors:** Estelle Martin, Wendy Tang, Cierra Briggs, Helena Hopson, Jose G. Juarez, Selene M. Garcia-Luna, Megan Wise de Valdez, Ismael E. Badillo-Vargas, Monica K. Borucki, Matthias Frank, Gabriel L. Hamer

**Affiliations:** 1 Department of Entomology, Texas A&M University, College Station, TX USA; 2 Department of Entomology and Nematology, University of Florida, Gainesville, FL USA; 3 Department of Science and Mathematics, Texas A&M University-San Antonio, San Antonio, TX USA; 4 Department of Entomology, Texas A&M AgriLife Research, Weslaco, TX USA; 5 Lawrence Livermore National Laboratory, Livermore, CA USA

## Abstract

South Texas has experienced local transmission of Zika virus and of other mosquito-borne viruses such as chikungunya virus and dengue virus in the last decades. Using a mosquito surveillance program in the Lower Rio Grande Valley (LRGV) and San Antonio, TX, from 2016 to 2018, we detected the presence of an insect-specific virus, cell fusing agent virus (CFAV), in the *Aedes aegypti* mosquito population. We tested 6,326 females and 1,249 males from the LRGV and 659 females from San Antonio for CFAV by RT-PCR using specific primers. Infection rates varied from 0 to 261 per 1,000 mosquitoes in the LRGV and 115 to 208 per 1,000 in San Antonio depending on the month of collection. Infection rates per 1,000 individuals appeared higher in females collected from BG Sentinel 2 traps compared to Autocidal Gravid Ovitraps, but the ratio of the percentage of infected pools did not differ by trap type. The natural viral load in individual males ranged from 1.25 x 10^2^ to 5.50 x 10^6^ RNA copies and in unfed females from 5.42 x 10^3^ to 8.70 x 10^6^ RNA copies. Gravid females were found to harbor fewer viral particles than males and unfed females.

## Introduction

In the continental United States, the states of Florida and Texas are emerging hotspots for *Aedes aegypti-*driven virus transmission. The Texas-Mexico border region has experienced local epidemics of dengue virus (DENV) in multiple communities [1–4]. In 2015, local transmission of chikungunya virus (CHIKV) occurred in Brownsville, TX, and 10 cases of Zika virus (ZIKV) were documented in South Texas by the Texas Department of State Health Services between 2016 and 2017 [5, 6].

Arbovirus disease transmission has been observed to vary from region to region [7, 8]. For example, DENV transmission in Tamaulipas, Mexico, during an epidemic period can amount to thousands of cases, while on the other side of the border in the Rio Grande Valley of Texas, United States, very few cases are recorded [4]. Disease dynamics are influenced by intrinsic and extrinsic factors among which the presence of microbes in the mosquito vector could enhance or reduce viral transmission [9, 10]. In the last decade, insect-specific viruses (ISVs) in mosquitoes have been shown to modulate the transmission of human pathogens (reviewed in reference [11]). However, ISVs can only infect and replicate in insect cells, causing no disease in humans [12]. The first ISV discovered was cell fusing agent virus (CFAV) (1974), which belongs to the classical ISVs [12]. Since its isolation from a natural mosquito population in Puerto Rico, United States [13], CFAV has been reported in multiple other countries, including Thailand [14], Indonesia [15], and Mexico [16]. While CFAV has been detected in field-collected mosquitoes, we are still lacking information regarding the prevalence and seasonality of infection in natural mosquito populations. In this study, we describe the infection rate of CFAV in *Ae. aegypti* populations from the Lower Rio Grande Valley (LRGV) and San Antonio, TX. Additionally, we evaluate the correlation of trap type, sex, and physiological status with the infection rate (IR) and the viral load in individual male and female *Ae. aegypti* mosquitoes.

## Materials and methods

### Mosquito sampling

**Fig. 1:**
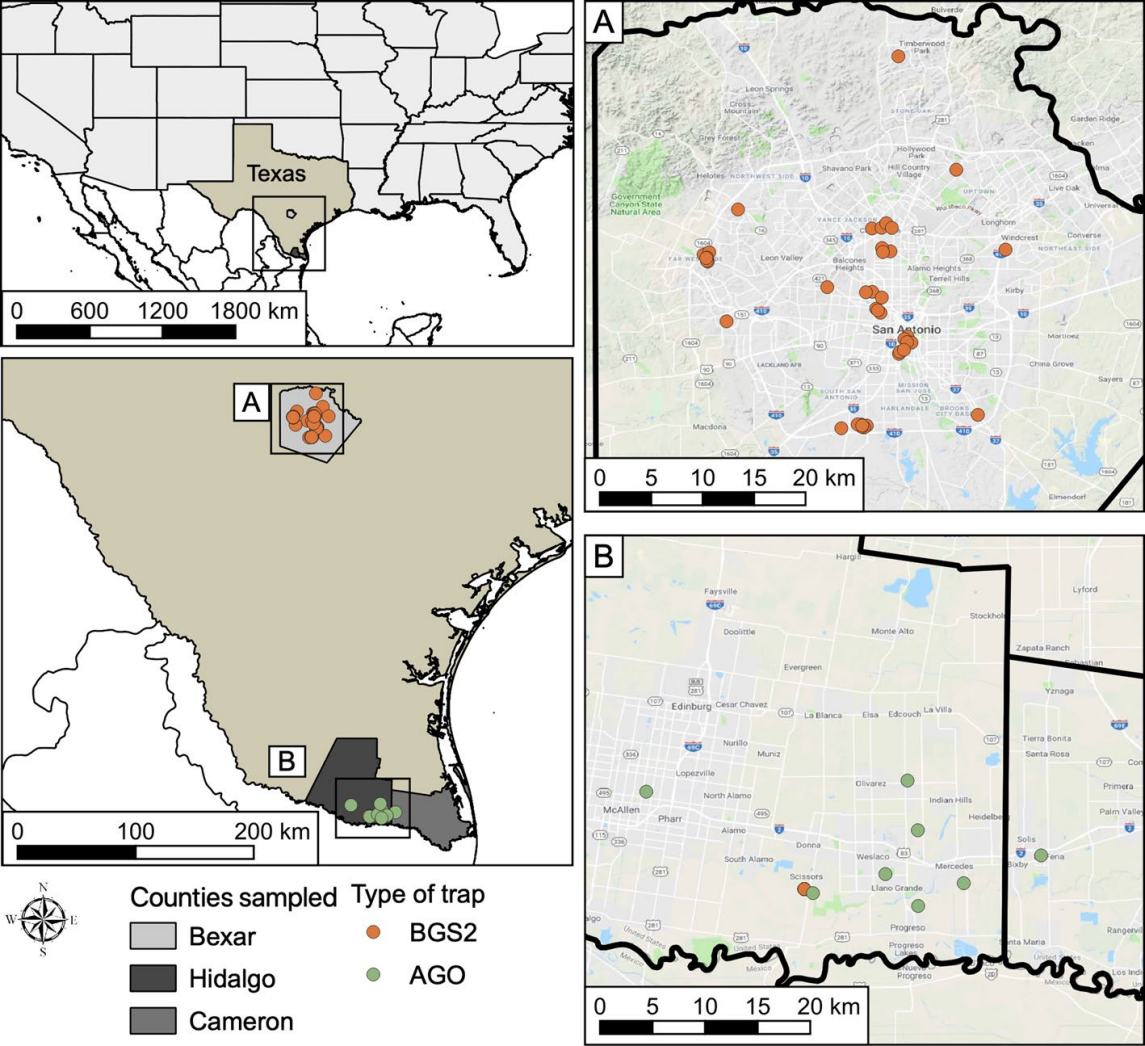
Study area and study sites for the collection of *Ae. aegypti* mosquitoes in the Lower Rio Grande Valley and San Antonio using AGO and BGS2 traps in 2017 to 2018. The map was made using QGIS 3.4.4 (https://qgis.org/en/site/). Map data: Google Maps, and with publicly available administrative boundaries (https://gadm.org/license.html)

Mosquito sampling using Autocidal Gravid Ovitraps (AGO) in the LRGV of South Texas was performed in seven communities (Balli, Cameron, Mesquite, Chapa, Christian Ct., La Vista and Rio Rico) located in Hidalgo County and one community (La Bonita) in Cameron County (Fig. 1) as described previously [6]. Briefly, AGOs baited with hay infusion were deployed inside and outside homes and sampled weekly. For mosquito sampling, BG Sentinel 2 traps (BGS2) with BG Lure (Biogents AG, Regensburg, Germany) were deployed in the community of La Piñata, Donna, TX, at 50 and 15 private residences in 2017 and 2018, respectively. In San Antonio, TX, BGS2 traps were deployed at 10 (2017) and 27 (2018) private residences across different ecological and socio-economical regions (Fig. 1). In both La Piñata and San Antonio, mosquitoes were sampled weekly (set in the morning and retrieved about 24 hours later). The species and sex of the mosquitoes from the AGO and BGS2 traps were determined and the mosquitoes from the BGS2 traps were additionally sorted by physiological status, and pools were stored at -80 °C until virus testing. To test for CFAV, we used a subset of *Ae. aegypti* that had been tested previously for ZIKV/CHIKV using a multiplex qRT-PCR assay [6]. Mosquito pools were homogenized in Hank’s buffer salt solution (HBSS) (Thermo Fisher Scientific, Waltham, MS) using a Tissuelyser II (QIAGEN, Hilden, Germany) and a stainless steel bead and then centrifuged for 1 min at 12,000 rpm. Subsequently, RNA was extracted from 250 µL of supernatant using a MagMAX™ CORE Nucleic Acid Purification Kit (Applied Biosystems, Foster City, California) following the manufacturer’s instructions. Individual mosquitoes used for the quantification of CFAV in the natural population were processed using the same protocol.

### Virus detection

In this study, we tested new samples from the LRGV and San Antonio (see above) using a conventional PCR assay with specific primers targeting a portion of the envelope gene of CFAV and a SuperScript^TM^ IV VILO ^TM^ Master Mix with ezDNase^TM^ Enzyme Kit (Thermo Fisher, Waltham, MA) to screen *Ae. aegypti* mosquito pools and individuals for CFAV [17]. Briefly, 0.5 µl of ezDNase and 0.5 µl of 10X ezDNAse Buffer were added to 4 µL of RNA to remove genomic DNA from the RNA preparation (2 min at 37 °C). Five microliters of the cleaned RNA was used for cDNA synthesis using SuperScript^TM^ IV VILO ^TM^ Master Mix. Two microliters of a 1:10 dilution of the cDNA was then amplified by PCR using the following cycling parameters: 94 °C for 3 min, 38 cycles of 94 °C for 30 s, 55 °C for 30 s, 72 °C for 1 min, and a final extension step at 72 °C for 8 min.

PCR products were run on 2 % agarose gel for 20 min, and positive samples were purified using Exo SAP-IT PCR Product Cleanup (Affymetrix, Santa Clara, CA) and sequenced. The sequences that were obtained were aligned, cleaned, and subjected to a BLAST search against the NCBI database using Geneious version 9.1.8. Sequences showing more than 98% identity were assigned to viral species. Additionally, positive samples were confirmed using a second PCR assay targeting the NS5 gene of CFAV [13]. The cycling protocol consisted of 95 °C for 2 min followed by 35 cycles of 94 °C for 30 s, 50 °C for 30 s, and final elongation at 72 °C for 1 min and 72 °C for 8 min. A subset of the amplicons were then sequenced by Eton Bioscience Inc. (San Diego, CA) using forward and reverse primers. To quantify the CFAV load in individual mosquitoes, we designed a gBlock (available upon request) to use as a standard for the qPCR analysis based on the PCR procedure described above [17] and a complete genome sequence of CFAV (accession no. NC_001564) in Geneious. Briefly, we used a Power SYBR Green RNA-To-Ct 1 Step Kit (Applied Biosystems, Foster City, CA) with 5 µL of RNA, 1 µL of each forward and reverse primer at 10 µM with the following cycling protocol: 48 °C for 30 min; 95 °C for 10 min, and 40 cycles at 95 °C for 15 s and 60 °C for 1 min.

### CFAV infection rates (IRs)

We estimated the CFAV IRs using the maximum-likelihood estimation (MLE) with 95% confidence intervals (CI) [18]. IRs were calculated per month for the mosquitoes collected from the LRGV using AGO traps and per week for the mosquito collected from the LRGV and San Antonio using BGS2 traps. To allow direct comparison, IRs for mosquitoes collected using AGO traps in the LRGV were also calculated per week of study.

### Statistical analysis

Statistical analysis was performed in GraphPad Prism version 7.0 for Mac (Graphpad Software, San Diego, California, USA). Variation in the mean virus load was tested using an unpaired *t*-test resulting in an exact *P*-value and 95% confidence interval. Comparison of the proportion of CFAV-infected pools was done using a chi-square (X^2^) test.

## Results

### Cell fusing agent virus detection and infection rate in *Ae. aegypti* collected in 2017-2018

**Fig. 2:**
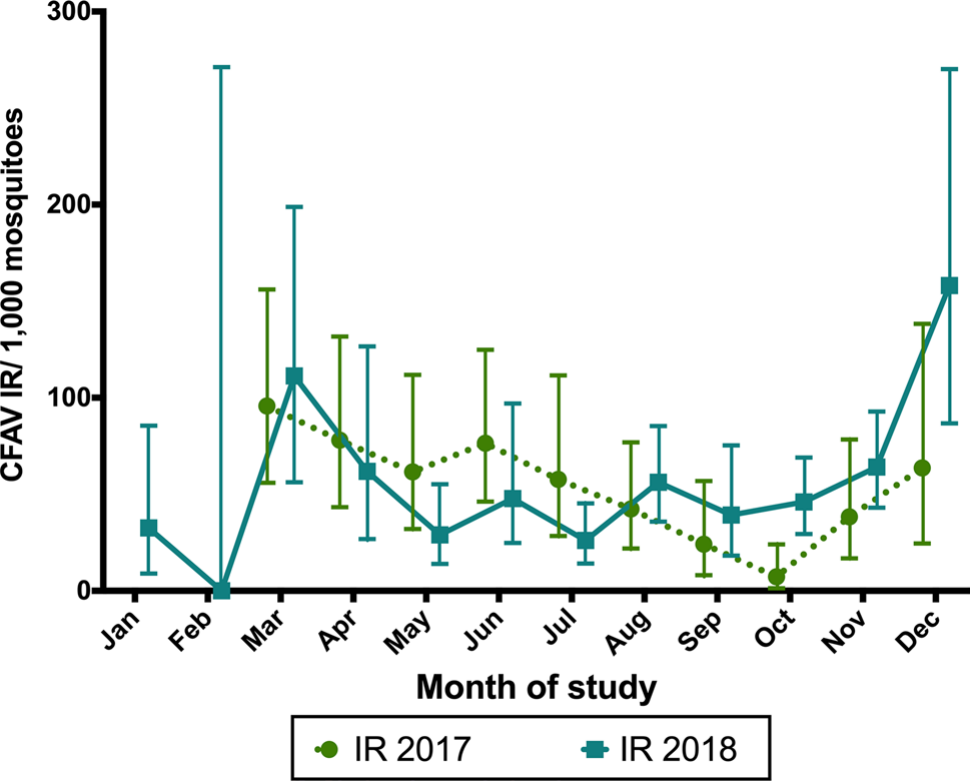
CFAV infection rates (IRs) per month of study in *Ae. aegypti* females from the LRGV collected using AGO traps in 2017 to 2018. The data points indicate the average CFAV IR per 1000 values observed, and the bars indicate the full range of CFAV IR per 1000 values observed

In the LRGV, 580 pools representing 5,215 individual *Ae. aegypti* mosquitoes were collected from AGO traps. Specifically, 205 pools (2,161 *Ae. aegypti* females) were captured from March to December 2017, and 375 pools (3,054 *Ae. aegypti* females) were collected from January to December 2018 (Table 1). The IR (per 1,000 individuals) per month ranged from 7.3 in October to 95.7 in March for 2017 and from 0 in February to 158.1 in December for 2018 (Fig. 2). CFAV infection was consistent throughout the months of sampling with no clear evidence of seasonal structure. A non-significant difference was observed in monthly IR between 2017 and 2018 (*p* > 0.05).

**Table 1:** Detection of CFAV in *Ae. aegypti* mosquitoes from the LRGV and San Antonio collected using AGO and BGS2 traps in 2017 and 2018

Location	Collection method	Year of collection	Sex	Number of mosquitoes	Number of mosquito pools	Number of positive pools
LRGV	AGO	2017 (Mar.-Dec.)	Female	2161	205	87
		2018 (Jan.-Dec.)	Female	3054	375	124
	BGS2	2018 (Sep.-Nov.)	Female	1111	399	119
	BGS2	2018 (Sep.-Nov.)	Male	1249	330	77
San Antonio	BGS2	2017 (Jun.)	Female	106	33	17
		2018 (May-Jul.)	Female	553	105	54

In San Antonio, 659 mosquitoes were collected from BGS2 traps and tested for CFAV infection. In 2017, we detected CFAV in June (IR = 208.6 [95% CI = 135.3-308]). Meanwhile, in 2018, CFAV was detected in May, June and July, with an IR of 151.1 (95% CI = 95.4-230.41), 182 (95% CI = 120.3-273.3) and 115.3 (95% CI = 67.70-188.8), respectively. No statistical difference in the proportion of CFAV-infected pools was observed between 2017 and 2018 for the month of June (*p* = 0.63).

### Influence of the trap type on the detection of CFAV in the LRGV

**Fig. 3:**
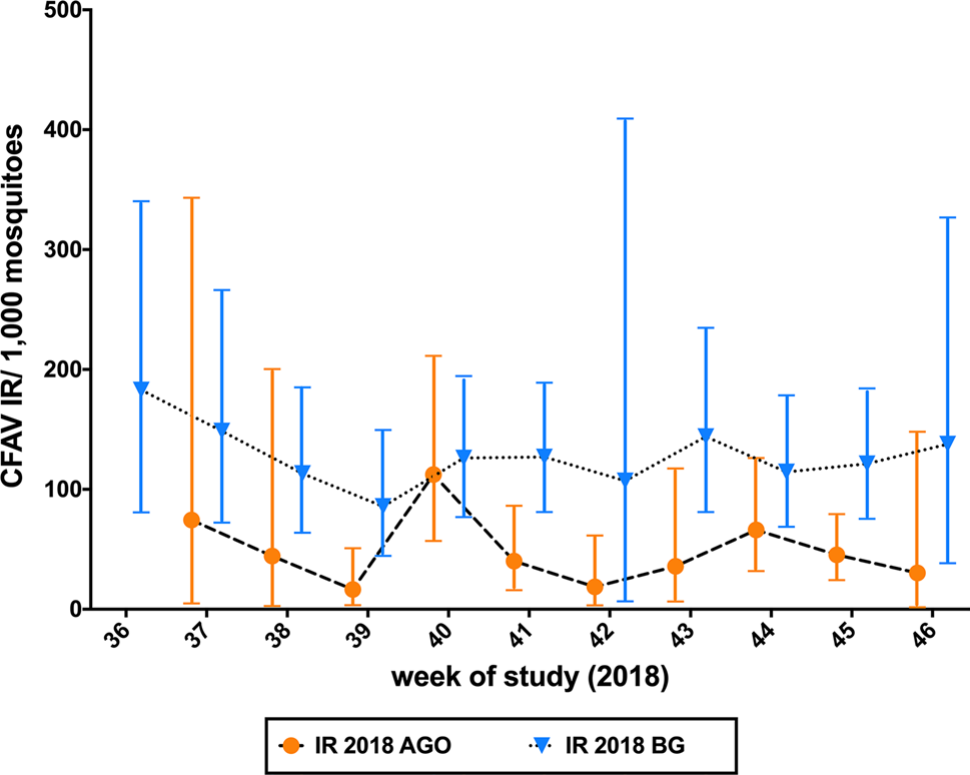
Comparison of infection rates (IRs) in *Ae. aegypti* from the LRGV collected in BGS2 and AGO traps in 2018. The data points indicate the average CFAV IR per 1000 values observed, and the bars indicate the full range of CFAV IR per 1000 values observed

In order to compare the CFAV IR according to trap type, 138 *Ae. aegypti* female pools representing 1,013 mosquitoes captured in AGO traps and 379 pools representing 1,076 females captured in BGS2 traps were tested. Both trap types were set up from epidemiological week (EW) 37-46 of 2018 in several communities of Hidalgo and Cameron counties, allowing a direct comparison. Forty of the pools collected using AGO traps and 113 pools collected using BGS2 traps were positive for CFAV. The AGO IR averaged 48.4 (95% CI = 16.4-112.3) and the BGS2 IR averaged 122.6 (95% CI = 85.8-148.6) (Fig. 3). Overall, the average IR was higher for the mosquitoes collected in BGS2 traps than for those collected in AGO traps (*p* < 0.0001). A week-to-week comparison showed a higher CFAV IR when mosquito pools were collected from BGS2 traps as compared to AGO traps, except for EW 40; however, the ratio of the number of CFAV-positive pools did not differ by trap type (*p* > 0.05).

### CFAV infection rate by sex and physiological status

**Fig. 4:**
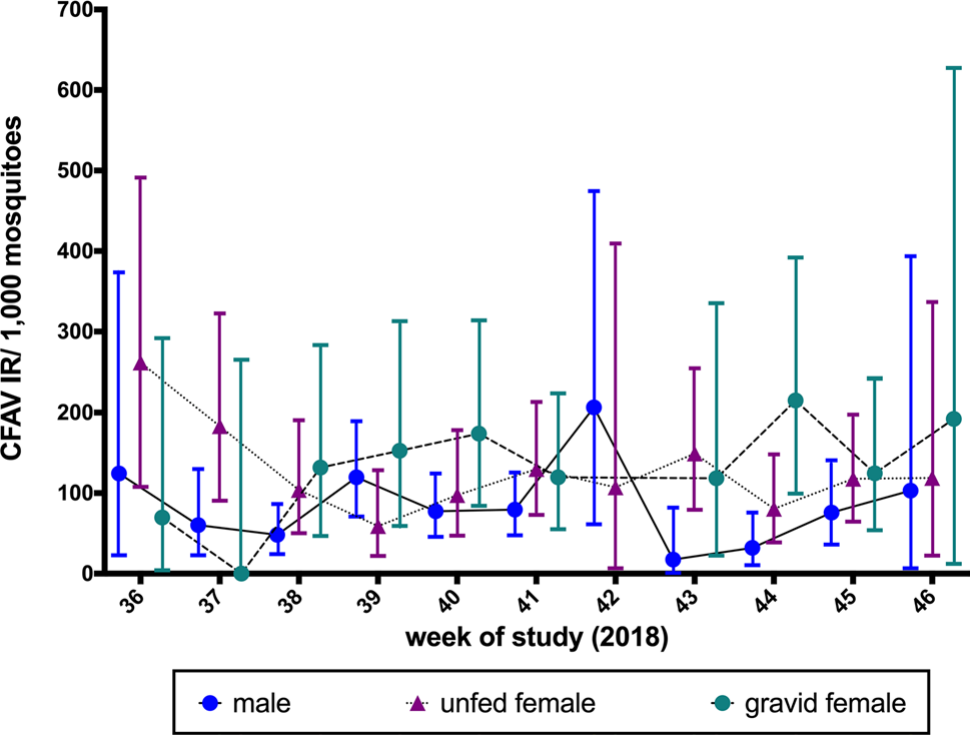
Comparison of infection rates (IRs) in male and female *Ae. aegypti* of different physiological stages captured in BGS2 traps in 2018. The data points indicate the average CFAV IR per 1000 values observed, and the bars indicate the full range of CFAV IR per 1000 values observed

*Ae. aegypti* mosquitoes collected during the EW 37 to 46 of 2018 with BGS2 traps in the LRGV were further analyzed by sex and physiological status. The CFAV IR ranged from 32 to 206 (in male mosquitoes average = 85.8 ± 17.7), from 58 to 261 in unfed females (average = 128 ± 16), and from 0 to 215 in gravid females (mean = 129 ± 19) (Fig. 4). While overall, the average IR in males was lower than in females, the difference was not significant (*p* = 0.08 compared to unfed females and *p* = 0.09 compared to fed females). Additionally, the proportion of infected pools was not statistically different between the different groups (*p* > 0.05).

### CFAV load in naturally infected individual mosquitoes

**Fig. 5:**
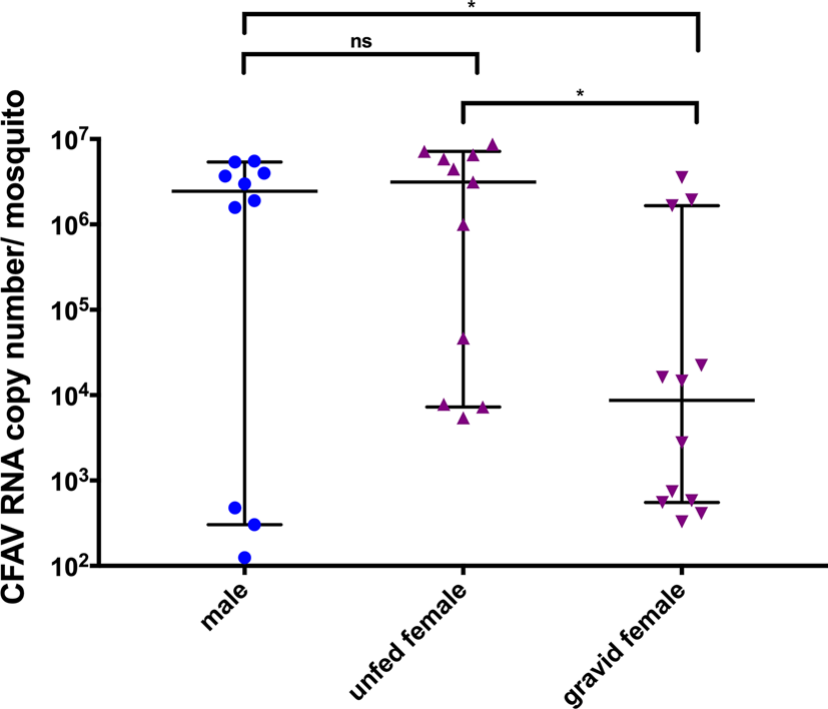
Median with 95% CI of the CFAV load in individual male and female *Ae. aegypti* of different physiological stages captured in BGS2 traps in 2018

The viral load in individual mosquitoes of different sex and physiological status collected from BGS2 traps in the LRGV was determined (Fig. 5). The viral titer is expressed as CFAV RNA genome equivalents. Overall, no statistical difference was observed between males (n = 10) and unfed females (n = 11; *p* = 0.49). The CFAV load for males ranged from 1.25 x 10^2^ to 5.50 x 10^6^ RNA copies per mosquito, and for females from 5.42 x 10^3^ to 8.70 x 10^6^ RNA copies per mosquito. A statistical difference was observed between gravid females (3.29 x 10^2^ to 3.53 x 10^6^ RNA copies per mosquito) and unfed females (*p* = 0.0134) as well as between gravid females and males (*p* = 0.015).

## Discussion

Although CFAV was discovered more than 40 years ago in an infected *Ae. aegypti* cell culture, its first detection in mosquito pools was in Puerto Rico in 2006 [13]. The virus was then detected in Thailand in 2007 and 2013 [14,1 9], Indonesia in 2009 [15], Mexico in 2011 [16], in Brazil and Australia in 2018 [20, 21] and in Africa in 2003 [22, 23]. In a previous study, we detected the presence of CFAV in two *Ae. aegypti* populations from Texas using a microarray developed at the Lawrence Livermore National Laboratory [24]. Although CFAV has been detected in the field on multiple occasions, few studies have provided an estimate of the infection rate in field populations of mosquitoes. Our study reveals the presence of CFAV throughout the year in *Ae. aegypti* collected using AGO traps in the LRGV (mean IR for 2017 = 54.5, mean IR for 2018 = 56.0) and at least from May to July for the San Antonio population collected using BGS2 traps (mean IR for 2017 = 208.6, mean IR for 2018 = 166.8). This study provides evidence that CFAV is widely distributed in Texas, although a direct comparison of IRs was not possible among the LRGV and San Antonio sites given that BGS2 trapping was performed at different time points. A previous study also documented CFAV in *Aedes* mosquitoes collected in Galveston, TX, in 2012 [25], adding to the overall picture of the geographic distribution of CFAV in Texas. Infection rates of CFAV were previously reported in an *Ae. aegypti* mosquito population from Thailand at a much lower rate, with an IR of 6.2 (n = 2110) in 2008 and 8.2 (n = 1944) in 2012 [14]. Differences in IRs could be due to the methodology used. In our study, mosquito homogenates were tested by PCR using gene-specific primers, whereas in the other study, mosquito homogenates were first screened for cytopathic effect in C6/36 cells, and only positive samples were tested by PCR using a pan-flavivirus assay followed by sequencing. Another insect-specific virus that infects *Culex* spp. mosquitoes, Culex flavivirus (CxFV), has received more research attention, and studies of infection rates and seasonality have been reported [26, 27]. In a study done in Iowa (USA), CxFV was not detected from May to June but occurred from July to October, while in Texas, the virus was detected from November to March with no detection during the months of April to August. A possible explanation for this seasonality relates to the overall number of mosquitoes collected and tested, which was reported for CxFV [28] and Aedes flavivirus (AeFV) [29] . In our study, an absence of CFAV was observed in the month of February 2018, when only nine pools (10 individual mosquitoes) were available for testing.

The infection rate of CFAV in the LRGV was compared using two trap types, AGO and BGS2. CFAV IRs from BGS2 traps were higher than those from AGO traps. IRs from BGS2 traps were 1.1 – 5.7 times higher than those from AGO traps. BGS2 traps were originally developed to target host-seeking females and contents of the trap must be inspected after 24 h of trapping [30, 31], while AGO traps mainly target gravid females for which the contents of the trap can be checked weekly [32–34]. While the lower rates of infection in the AGO-collected mosquito pools could have been due to degradation of the viral RNA after a week of trapping, it is also possible that the physiological status of the females collected in the different traps influences the IR. In our study, gravid females did indeed harbor fewer viral particles than unfed females. This represents an unique observation, because very few studies have documented arboviral loads in naturally infected females from the field, in part because infected individuals are rarely detected and as a result are typically tested in pools to reduce costs [35]. However, there has been one report of the CxFV infection loads in a *Culex pipiens* colony naturally infected in Colorado [36]. It has been hypothesized that traps targeting gravid females should have a higher viral IR than traps targeting host-seeking females, given that gravid females are more likely to be on average older than unfed females and more likely to have had at least one bloodmeal [37]. While some studies comparing the West Nile virus IRs between gravid and unfed female *Culex* spp. mosquitoes did confirm this hypothesis [38, 39], other studies found no statistical differences or opposite results [40]. One possible explanation for the reduced viral loads in gravid females could be that the immune response in gravid females is different from that in unfed females. Variation in the mosquito immune response has been demonstrated following the ingestion of the bloodmeal or the production of eggs [41–43]. Also, we found variation in the individual virus load, with some mosquitoes harboring a low number of genome equivalent while others had higher copy numbers. This reflects the natural variation in the involvement of mosquitoes when mounting an immune response to CFAV infection. While AGO traps have been developed as mosquito population surveillance and control tools, we demonstrated that samples from this trap can also be used for viral surveillance. Due to its cost efficiency and reduced equipment and labor requirements, this study suggests that the AGO trap is a viable option for monitoring vector populations for viral infection, especially in remote locations or developing countries.

CFAV was detected in both male and female field-collected mosquitoes, confirming the vertical transmission of this virus [13, 14, 17], a common mechanism for maintenance of ISVs such as CxFV [44, 45], AeFV [46] and Kamiti River virus [47].

To date, only one report has shown coinfection with CFAV and DENV (DENV-4) in field-collected mosquitoes [19]. In that study, mosquitoes (n = 93) were collected in the homes of DENV-infected patients, which might have increased the probability of detecting coinfection. No evidence of coinfection between CFAV and ZIKV or CHIKV has been observed. Our samples were tested previously for ZIKV and CHIKV during an arbovirus surveillance study from 2016 to 2018 and none were positive [6]. Several ISVs have demonstrated their importance in the modulation of the transmission of certain pathogens. For example, CxFV and Nhumirim virus both have the capacity to modulate WNV transmission by *Culex* mosquitoes [36, 48–50]. The ability of CFAV to influence DENV replication has been demonstrated *in vitro* [51, 52], and its ability to influence arboviral disease transmission has been demonstrated *in vivo* for ZIKV and DENV by intrathoracic injection. In those studies, a reduction in arbovirus transmission was observed. Hence, if these observations are confirmed in the context of a natural infection (as opposed to intrathoracic injection), the low number of cases observed in the LRGV during the 2016-2017 ZIKV outbreak could be at least partially explained by the presence of CFAV in the *Ae. aegypti* population in that area. Therefore, additional studies on the geographic prevalence of CFAV in *Ae. aegypti* populations in Texas could help with the prediction of emergence of arboviral disease in the region.
